# Sixth Annual Meeting of the Illinois State Medical Society

**Published:** 1856-07

**Authors:** N. S. Davis

**Affiliations:** Sec. Ill. State Med. Society


### MEDICAL SOCIETIES.

*Sixth Annual Meeting of the Illinois State Medical Society, held in Vandalia, June* 3 and 4, 1856.

MORNING SESSION, June 3.

The members of the Society assembled in the Old State House, and were called to order at 11 o'clock, by the President, Dr. N. S. Davis, of Chicago.

Dr. Stearns, Chairman of the Committee of Arrangements, reported the names of the following delegates and members as having been registered by the Committee, viz.:—

DELEGATES.

Dr. H. Noble, for McLean County Medical Society.

" T. R. Edmiston, for DeWitt County Medical Society.

" P. H. Lawrence, for Christian " " "

" J. W. Freer, for Cook " " "

" J. N. Cameron, } for Esculapian Medical Society.

" Thos. D. Washburn,

" James M. Steel,

" D. N. Moore, } for Union Medical Society.

" Wm. White,

" J. V. Z. Blaney, } for Rush Medical College.

" Wm. B. Herrick,

PERMANENT MEMBERS.

Dr. Samuel Thompson, of Albion, Edwards County.
" A. H. Luce, of Bloomington, McLean "
" S. W. Noble, of LeRoy, " "
" S. T. Trowbridge, Decatur, Macon "
" W. A. Hillis, of Hillisboro, Montgomery "
" N. S. Davis, of Chicago, Cook "
" F. B. Haller, of Vandalia, Fayette "
" A. D. Stearns, " " "

The President informed the Society that neither of the Secretaries were present, when, on motion of Dr. H. Noble, Dr. J. W. Freer, of Chicago, was unanimously elected Secretary *pro tem.*

The President announced the regular business in order to be the election of officers of the Society for the coming year.

On motion of Dr. W. B. Herrick, the appointment of a committee to nominate officers was postponed until the commencement of the afternoon session.

The Secretary *pro tem.* read a communication from the Secretary, Dr. E. Andrew, of Peoria, accompanied by his report as Chairman of the Committee of Arrangements. According to this report, the expense of publishing the Transactions of last year was $117 00, all of which the Secretary had paid from his own funds, having received nothing from the Society.

After supplying the members with copies of the Transactions, 350 copies remained in his hands, subject to the order of the Society.

The Treasurer, Dr. J. V. Z. Blaney, explained that he had received no order from the Secretary for money, nor indeed any communication from him on any subject since the last meeting of the Society.

He also presented the following report, viz.:—

J. V. Z. BLANEY, TREASURER,
To ILLINOIS STATE MEDICAL SOCIETY, DR.

| | |
|---|---|
| To amount collected of members at the annual meeting in June, 1855, as by accompanying list, | $96 00 |
| CR. | |
| By Cash paid June 6th, 1855, to N. S. Davis, late Treasurer, balance due him as by his last report, | 56 00 |
| Balance on hand at this date, June 3d, 1856, | 40 00 |
| | $96 00 |

In addition to the above, the Treasurer holds, subject to the order of the Prize Committee, $26 00, and the offer of $20 00 more from Dr. N. S. Davis, whenever the committee make an award.

On motion, the reports of the Secretary and the Treasurer were accepted and referred to a committee of three, with instructions that the committee audit the same, and also recommend the sum to be fixed as the annual assessment for the present year. The President appointed Drs. Samuel Thompson, H. Noble, and Dr. W. B. Herrick, said committee.

On motion, the Society adjourned until 2 o'clock, P.M.

AFTERNOON SESSION.

The Society was called to order at 2 o'clock P.M., the President, Dr. N. S. Davis, in the chair.

On motion of Dr. W. B. Herrick, the regular order of business was suspended for the purpose of electing permanent members; whereupon the following gentlemen were duly nominated and elected by unanimous votes:—

Dr. D. N. Moore, of Clinton County, proposed by Dr. F. B. Haller; Dr. T. S. Brooks, of Greenville, proposed by Dr. W. B. Herrick; Dr. H. Shoemaker, of Columbia, proposed by Dr. S. Thompson; Dr. T. J. Cornell, of Waterloo, proposed by Dr. S. Thompson; Dr. W. J. Gibbs, of ———, proposed by Dr. W. B. Herrick; Dr. W. J. Chenowith, of Decatur, proposed by Dr. S. T. Trowbridge; Dr. Ira B. Curtis, of Decatur, proposed by Dr. S. T. Trowbridge; Dr. E. W. Moore, of Decatur, proposed by Dr. S. Thompson; Dr. J. Barbre, of Louisville, proposed by Dr. F. B. Haller; Dr. Wm. Noecker, of Monticello, proposed by Dr. S. T. Trowbridge; Dr. Vermillia, of Chicago, proposed by Dr. J. V. Z. Blaney; Dr. J. H. Hollister, of Chicago, proposed by Dr. J. W. Freer; Dr. Edmund Andrews, of Chicago, proposed by Dr. W. B. Herrick; Dr. J. M. Williamson, of Lovington, proposed by Dr. W. B. Herrick; Dr. E. W. Boothe, of Vanburensburgh, proposed by Dr. F. B. Haller; Dr. F. R. Payne, of Marshall, proposed by Dr. S. Thompson; Dr. H. R. Payne, of Marshall, proposed by Dr. S. Thompson; Dr. Hiram Nance, of Lafayette, proposed by Dr. S. Thompson; Dr. S. Hubbard, of Bloomington, proposed by Dr. A. H. Luce; Dr. Lee Smith, of Hudson, proposed by Dr. A. H. Luce; Dr. S. McBride, of Decatur, proposed by Dr. W. Moore; Dr. Wm. Chambers, of Charleston, proposed by Dr. J. N. Cameron; Dr. W. H. Burns, of Richrum, proposed by Dr. J. N. Cameron; Dr. H. Barber, of Richrum, proposed by Dr. J. N. Cameron; Dr. Wm. Hill, of Salem, proposed by Dr. W. White; Dr. Jayne, of Nashville, proposed by Dr. W. A. Hillis.

Dr. S. Thompson, from the Special Committee, to whom was referred the reports of Treasurer and Secretaries, reported that they had examined the same and found them correct. The Committee also recommended the adoption of the following resolutions:—

*Resolved*, That an assessment of three dollars be made on all the present and past members of this Society, whose names appear on the different volumes of Transactions of this Society. Also, that the Secretary be instructed to inform them of the same, and that the payment for this one year shall reinstate them in full membership.

*Resolved*, That the Treasurer be authorized to refund to Dr. Andrew, of Peoria, late Secretary, the amount set forth in his report as having been advanced for publishing the Transactions of the Society, at as early a period as possible.

On motion, the report of the Committee was accepted and the resolutions adopted.

The Society took a recess of ten minutes, to enable the members from each County to select from their own number, one to constitute a committee to nominate officers for the ensuing year.

The President again called the Society to order, and the Nominating Committee was announced as follows:—

Dr. A. H. Luce, of McLean County.
" T. R. Edmiston, of DeWitt County.
" J. R. Lawrence, of Christian "
" J. W. Freer, of Cook "
" S. T. Trowbridge, of Macon "
" W. A. Hillis, of Montgomery "
" D. N. Moore, of Clinton "
" W. White, of Marion "
" J. Barbre, of Clay "
" F. S. Brooks, of Bond "
" J. N. Cameron, of Coles "
" J. M. Steel, of Edgar "
" S. Thompson, of Edwards "
" J. M. Williamson, of Moultrie "
" F. B. Haller, of Fayette "

The Committee retired, and after consultation, reported through their Chairman as follows:—

*President*—Dr. H. Noble.

*Vice-Presidents*—Dr. T. D. Washburn; Dr. F. B. Haller.

*Secretaries*—Dr. N. S. Davis, of Chicago; Dr. H. A. Johnson, of Chicago.

*Treasurer*—J. V. Z. Blaney, of Chicago.

On motion, the report was accepted, and the candidates named therein unanimously elected officers of the Society for the ensuing year.

The President appointed a committee, consisting of Drs. Luce, Blaney, and S. W. Noble, to conduct the newly elected officers to their respective places.

Dr. H. Noble, on being conducted to the chair, thanked the Society for the honor conferred on him, and announced the regular order of business to be the hearing of reports from the Standing Committees.

Dr. S. Thompson, of Albion, Chairman of the Committee on Practical Medicine, made a lengthy report, which was listened to with much interest.

On motion of Dr. Luce, of Bloomington, the report from the Committee on Practical Medicine, &c., was accepted and referred to the Committee on Publication.

The standing committees on Surgery, Obstetrics, and Drugs and Medicines, being called in order made no reports.

On motion of Dr. J. V. Z. Blaney, the Committee on Drugs and Medicines, Dr. H. A. Johnson, of Chicago, Chairman, was continued another year.

Dr. J. V. Z. Blaney, special committee on the Toxicology of the Alkaloids, reported that his investigation of the subject was yet incomplete; and, on motion of Dr. S. Thompson, the committee was continued another year.

Dr. D. Prince, of Jacksonville, special committee on Orthopœdic Surgery, being absent, his report on that subject was read by the Secretary; accepted, and referred to the Committee on Publication.

A letter was read from Dr. F. K. Bailey, of Joliet, special committee on Congestive Intermittents, apologizing for his absence and inability to report at the present meeting.

On motion, Dr. Bailey was continued a committee on the same subject another year.

Dr. A. S. McArthur, of Joliet, special committee on the Physiological Explanation of Counter-Irritation, not being present, he was, on motion of Dr. J. V. Z. Blaney, continued another year.

Dr. N. S. Davis, of Chicago, special committee on the Alterations of the Blood in Continued Fevers, stated that he could be prepared to make a partial report to-morrow. But, after some explanations, on motion of Dr. Thompson, the committee was continued another year.

Dr. J. V. Z. Blaney, from the committee appointed at the last annual meeting to memorialize the legislature in favor of further provisions for the accommodation of the insane, reported that there had been no meeting of the legislature since the appointment of the committee, and consequently that they had hitherto been unable to perform the duties assigned them.

On motion, the committee were continued another year.

Dr. Blaney also called the attention of the Society to the subject of procuring laws for the registration of births, marriages, and deaths; asking for the appointment of a committee to act with the committee of the American Medical Association appointed to procure the enactment of uniform laws on the subject in all the States.

Dr. S. Thompson presented a resolution from the Esculapian Society on the same subject, which, after being amended, was adopted as follows:—

*Resolved*, That a committee, of one from each County, be appointed, Prof. Blaney, of Chicago, being chairman, to memorialize the legislature to pass a law for the registration of births, marriages, and deaths, in this State; and that the committee be requested to co-operate with the committee of the American Medical Association on the same subject.

The President announced the following as the committee under this resolution, viz.:—

Drs. S. T. Trowbridge, of Macon County; S. W. Noble, of McLean; S. Thompson, of Edwards; F. B. Haller, of Fayette, A. L. Keller, of Moultrie; C. Goodbrake, of DeWitt; W. A. Hillis, of Montgomery; S. W. Thompson, of Clay; G. N. Logan, of Crawford; J. F. Daggett, of Will; H. R. Payne, of Clark; Boal, of Marshall; W. S. Maus, of Tazewell; E. C. Banks, of Coles; S. Long, of Sangamon; J. D. Arnold, of Peoria; J. C. Fitts, of Fulton; B. F. Stevenson, of Menard; J. B. Samuel, of Green; W. W. Welch, of LaSalle; H. Shoemaker, of Monroe; J. W. Spaulding, of Knox; H. Nance, of Stark; S. A. Paddock, of Bureau; D. Lyman, of Winnebago.

On motion of Dr. N. S. Davis, a committee of five was appointed to select a place for the next annual meeting, and also to fill up the Standing Committees for the ensuing year.

The President appointed Drs. Herrick, Thompson, Luce, Washburn, and Edmiston said committee,

On motion, the Society adjourned to 9 o'clock A. M., to-morrow.

WEDNESDAY, June 4.

The President, Dr. H. Noble, called the Society to order at 9 o'clock A. M.

The Secretary, Dr. N. S. Davis, read the minutes of the last meeting, and they were approved.

Dr. S. Thompson offered the following amendment to the Constitution, which lies on the table until the next annual meeting, viz.:—

*Resolved*, That the Society shall choose one Permanent Secretary, to hold office during the pleasure of the Society, and whose duty it shall be to keep all the records, books, and papers of the Society; and one Assistant Secretary, to be elected annually from the place where the next succeeding meeting is to be held. The Permanent Secretary, when not able to attend any given meeting, shall furnish the Assistant with so much of the records and papers as are necessary for the accommodation of that meeting, which, together with the proceedings of the said meeting, shall be returned to him as soon as practicable after the adjournment of the same.

On motion of Dr. F. B. Haller, Dr. J. Campbell, of Mulberry Grove, Bond County, and any other physicians in good standing in the profession who may be present, were invited to take part in the meeting as members by invitation.

Dr. A. H. Luce, from the special committee to select a place for the next Annual Meeting, &c., reported as follows:—

Place for the next Annual Meeting, *Chicago*.

*Committee of Arrangements.*—Dr. J. H. Hollister, Dr. DeLaskie Miller, Dr. J. Bloodgood, Dr. Chas. G. Smith, Dr. Henry Parker, all of Chicago.

*Committee on Practical Medicine*—Dr. C. N. Andrews, of Rockford; Dr. F. R. Payne, of Marshall; Dr. S. T. Trowbridge, of Decatur.

*Committee on Surgery*—Dr. J. W. Freer, of Chicago; Dr. David Prince, of Jacksonville; Dr. A. H. Luce, of Bloomington.

*Committee on Obstetrics*—Dr. J. C. Frye, of Peoria; Dr. C. Goodbrake, of Clinton; Dr. Wm. Chamberlain, of Toulon.

*Committee on Drugs and Medicines*—Dr. H. A. Johnson, of Chicago; Dr. J. Blount, of Rockford; Dr. S. W. Thompson, Clay County.

Also, the following special committees, viz.:—

Dr. C. Goodbrake, of Clinton, on the Medicinal Properties of the Asclepias Tuberosa.

Dr. G. W. Moore, of Decatur, on Anemia and Chlorosis.

On motion of Dr. Hillis, seconded by Dr. White, the foregoing report was accepted and adopted as a whole.

Dr. Trowbridge, in behalf of the citizens and profession of Decatur, invited the Society to hold its annual meeting for 1858 in that place.

Dr. S. Thompson, called the attention of the Society to the practice of selling patent medicines and nostrums, and the publishing of unprofessional matter in the newspapers by members of the Society. He alluded to Dr. A. G. Lawton, of Lasalle, and closed by a motion that he be expelled from the Society. The motion was seconded and passed by a unanimous vote.

Dr. Blaney offered the following resolution, which was adopted:—

*Resolved*, That the Committee on Publication be requested to publish, in pamphlet form, 1,000 copies of the Constitution of the Society, the By-Laws, and Code of Ethics, as now in force, together with such explanatory or declaratory resolutions as may be found in the proceedings of the Society, and forward a copy of the same to each member of the Society.

Dr. Haskell, who had been alluded to by Dr. Thompson as implicated in selling nostrums, had sent a request that he might withdraw from the Society; and, on motion of Dr. Washburn, his request was granted.

On motion of Dr. S. W. Noble, the Society proceeded to elect twelve delegates to the next Annual Meeting of the American Medical Association, to be held in Nashville, Tenn., on the first Tuesday in May, 1857, as follows:—Drs. A. H. Luce, of Bloomington, S. Thompson, of Albion, J. V. Z. Blaney, of Chicago, T. R. Edmiston, of Clinton, J. M. Steel, of Grand View, S. T. Trowbridge, of Decatur, S. W. Noble, of Leroy, J. M. Williamson, of Lovington, F. B. Haller, of Vandalia, W. A. Hillis, of Hillsboro, J. N. Cameron, of Charleston, and Thos. Hall, of Toulon.

On motion of Dr. Luce, the Society authorized the Secretary to publish the Transactions in the *North-Western Medical and Surgical Journal*, if himself and the Treasurer should deem it expedient.

Dr. Blaney offered the following, which was adopted, as an addition to the By-Laws of the Society, viz.:—

*Resolved*, That the Chairman of all committees, the Secretary and Treasurer, each have authority to have printed such circulars as may be required for the efficient performance of their duties; and that they present their accounts for the same and for postage, to the meeting at which they make their reports.

On motion of Dr. Blaney, the resolution adopted at the last annual meeting, in relation to the initiation fees of new members, was continued in force for the present year.

Dr. N. S. Davis offered the following resolution, which was adopted:—

*Resolved*, That a committee of three be appointed on Prize Essay, authorized to offer a premium of $50 for the best Essay on some medical subject, to be presented under the usual regulations.

The President appointed Drs. J. V. Z. Blaney, T. D. Washburn, and S. Thompson, the Committee on Prize Essay.

On motion of S. Thompson, the Committee of Arrangements were requested to consider the propriety of issuing annual cards of membership, with power to act.

Dr. N. S. Davis, the retiring President, then read his valedictory address.

Dr. Washburn offered the following, which was adopted:—

*Resolved*, That this Society have listened with great pleasure and satisfaction to the very able and interesting valedictory address of Prof. N. S. Davis, on the Influence of Alcohol on Tuberculosis.

Dr. Luce offered the following, which was seconded by Dr. Trowbridge, and adopted, viz.:—

*Resolved*, That the thanks of the Society be tendered to Dr. Davis for his very able valedictory address, and that a copy of the same be requested for publication in the Transactions of the Society.

On motion of Dr. T. D. Washburn, the following was adopted:

*Resolved*, That the thanks of the Society are hereby tendered to the Committee of Arrangements, for the efficient and faithful manner in which they have discharged their duties.

Dr. White offered the following resolution, which was unanimously adopted:—

*Resolved*, That we, the members of this Society, tender our sincere thanks to the Masonic fraternity of Vandalia, for the use of the room and furniture in which we have held our meetings; and that the chairman of the late Committee of Arrangements be requested to present a copy of this resolution to the Master of the Masonic Lodge.

The following was offered by Dr. Luce, and also unanimously adopted:—

*Resolved*, That the thanks of the Society are hereby tendered to the citizens of Vandalia for their hospitality and kind accommodation of its members during its present annual session.

On motion of Dr. S. Thompson, the Society adjourned *sine die*.

N. S. DAVIS, *Sec. Ill. State Med. Society.*

